# Incompatible: end-of-life care and health economics

**DOI:** 10.1136/bmjspcare-2020-002388

**Published:** 2021-03-08

**Authors:** Katharina Diernberger, Bethany Shinkins, Peter Hall, Stein Kaasa, Marie Fallon

**Affiliations:** 1 Cancer Research UK, Institute of Genetics and Molecular Medicine, University of Edinburgh Western General Hospital, Edinburgh, UK; 2 Faculty of Medicine and Health, Academic Unit of Health Economics, University of Leeds, Leeds, UK; 3 Institute for Clinical Medicine, Faculty of Medicine, University of Oslo, Oslo, Norway; 4 Department of Palliative Medicine, University of Edinburgh, Western General Hospital, Edinburgh, UK

**Keywords:** service evaluation, end of life care, clinical decisions

When it comes to death, the statistics are stark. 100% of us will die. The question is what are we all going to do about that? How are we going to create conﬁdence in the care that we may need?^1^

During the last year of life, major healthcare resources are spent, not only in lifetime monetary terms, but also on professional time. Reflecting on this quote, it seems counterintuitive that health economics could play a major role in tackling the main challenges in end of life care. However, the escalating cost of healthcare, combined with an ever-increasing range of therapeutic and patient management options, has brought difficult budget allocation decisions to the fore.

## What is the value of health care?

The value of healthcare can be considered as what is gained relative to what is lost. In our context, there are three value dimensions:

1. Population: how well assets are distributed to different subgroups in society (equity in resource distribution).
2. Technical: how well resources are used for outcomes for all people in need in the population (improving quality and safety of services).
3. Personal: how well the outcome relates to the values of each individual (understanding what matters most to the patient).

Contrary to popular misconception, value is not the same as quality of care or how much money is spent. High-quality care to the wrong patient or at the wrong time (or in the wrong place), is still low value. Similarly, better value is not necessarily achieved by more money. Nevertheless, even to the right person at the right time, it will still have an inevitable cost

However, maximising value in healthcare resources requires understanding both what we seek to achieve and the effectiveness of the means to achieve it; this is the purpose of health economics.

People used to a universal healthcare system may struggle to see the value of healthcare, rather than perceive it as a basic right and rarely question where these resources originate.

## What is health economics?

*‘Economics is a science which studies human behaviour as a relationship between ends and scarce means which have alternative use*’. 2 Thus, economics is a science of choice. Health economics is therefore the science of choice within the healthcare context. The aim is to distribute a constrained health budget to maximise overall population health.

A key concept of economic theory is ‘opportunity cost’, defined as ‘(*t)he value of forgone benefit which could be obtained from a resource in its next best alternative use’*.^3^ Fundamentally, money spent on a certain intervention/treatment/drug cannot be spent on something else—even though that may also have had a beneficial outcome. In reality, healthcare systems are so complex that the opportunity cost is typically NOT identifiable, that is, we do not know what other healthcare intervention we may have displaced.

The economic evaluation framework quantifies the pros and cons of specific health interventions and balances them against the cost (which might be to the system or the individual). With such a framework, we can therefore reduce ‘waste’ by identifying and exchanging treatments that may be of minimal benefit for more effective ones.

## Health economics and palliative care

The care of terminal or highly symptomatic disease is expensive, with both a financial and capacity strain on individuals and local and national health systems globally. This is exacerbated by a demographic shift in age distribution; people live longer with more health needs in later life. Medical and technological advances expand treatment options, many at great cost. For example, new anticancer treatments, like immunotherapies and targeted anticancer therapies improve progression-free survival, sometimes overall survival but significantly increase costs at end of life. Drugs recently approved by the National Institute for Health and Care Excellence (NICE) for poor prognosis cancers typically cost about an extra £50 000 for each quality of life-adjusted year (QALY) gained—a composite measure of individual quality of life and survival.

As the healthcare budget is constrained, hard choices must be made. Real patient care has numerous challenges and limits on finance and appropriate healthcare worker time. One important example is community care, which is largely dependent on the number of available informal carers. Prioritising between this type of care and palliative anticancer treatments is an inherent tension. Health Economic evaluations assist decision making on a larger scale, like the choice between additional palliative care beds or new drugs and more intensive care.

## Problems of health economics in palliative care

As health economics informs decision making, influencing the quantity, quality and sustainability of healthcare resources, it is imperative this methodology is applied to the highest possible standards. Within the UK, a standard approach to compare the cost-effectiveness of interventions has been established by decision makers like NICE. It relies on the costs to the National Health Service (NHS) and social care balanced against differences in QALYs.

For several reasons, this approach falls short when evaluating interventions at the end of life.

- First, a significant proportion of the important costs are likely to be incurred outside of the NHS, the charitable sector, the welfare state or the individual and their families and/or carers. These currently fall outside of a NICE standard economic evaluation.
- Second, it is inaccurate to measure patient benefits because the improved function is not expected. The standard methods for quantifying health outcomes are problematic in end-of-life care as the patient needs/focus are different than in those expected to improve.
- Third, the QALY is the recommended tool for capturing health outcomes across different clinical and disease areas. However, the ability of the QALY to capture aspects of health important to patients in an end-of-life context has been questioned given the aim at that juncture is neither improved survival nor function. The aims are to prevent and treat symptoms, preserve function, shared decision making and family care.

New research programmes are testing different strategies of better capturing patients’ priorities at the end of life like the burden of illness measures or palliative-specific quality of life measurements such as Investigative Choice Experiments of CAPablility measures.

## Why is it urgent?

Worldwide, the financial cost to an individual with severe illness is significant. In the USA, the risk of bankruptcy increases by 250% with a cancer diagnosis.^4^ Even in the UK where healthcare is free at delivery, those with a cancer diagnosis were found, on a monthly average, to be £570 poorer.^5^

In the UK, most people die in hospital, despite it being the least preferred location.^6^ Many may have unnecessary clinical interventions unlikely to impact quality and/or length of life.^7^ Hospital care is expensive but comprehensive palliative care at home may also be costly.

Tailored end-of-life care integrated into public healthcare reduces emergency hospital and intensive care unit admissions and length of hospital stay.^8 9^ A more personalised approach therefore has great potential to avoid unnecessary resource use while simultaneously benefitting the patient.

In the UK, all these issues are being tackled by a new national strategy to redesign palliative care services. But is it a need to prioritise, for example, between expensive new drugs with limited life prolongation and little evidence of improved symptom management or a basic human right to good end of life care? In line with national ambitions for personalised care, advanced care planning is at the heart of this strategy, where patients should have realistic high-quality choices at the end of life.^1 10^ The effectiveness of sustainable integrated palliative care programmes^11^—including the funding of end-of-life services—is well documented^12^ and it may be best to prioritise such interventions in a public health system.

How much a society is able and prepared to spend on those who are sick and face approaching death may differ; views will vary across (and within) continents and countries and between faith and value systems.^13^

The goal therefore should be to reduce the financial burden of care of the dying on the healthcare system without compromising the level of care or a person’s quality of life. If the palliative care clinical community accepts available resources are constrained, then extensive work is necessary to better understand the value at the end of life.
